# Diffuse Reflectance Spectroscopy for Black Carbon Screening of Agricultural Soils under Industrial Anthropopressure

**DOI:** 10.3390/molecules27217334

**Published:** 2022-10-28

**Authors:** Guillaume Debaene, Aleksandra Ukalska-Jaruga, Bożena Smreczak, Ewa Papierowska

**Affiliations:** 1 Department of Soil Science Erosion and Land Protection, Institute of Soil Science and Plant Cultivation—State Research Institute, Czartoryskich 8, 24-100 Puławy, Poland; 2 Institute of Environmental Engineering, Water Center, Warsaw University of Life Sciences, Nowoursynowska 159, 02-776 Warsaw, Poland

**Keywords:** black carbon, soil organic carbon, VIS-NIR, SVM, PLS-SVM classifier

## Abstract

Visible and near-infrared spectroscopy (VIS-NIRS) is a fast and simple method increasingly used in soil science. This study aimed to investigate VIS-NIRS applicability to predict soil black carbon (BC) content and the method’s suitability for rapid BC-level screening. Forty-three soil samples were collected in an agricultural area remaining under strong industrial impact. Soil texture, pH, total nitrogen (N_tot_) and total carbon (C_tot_), soil organic carbon (SOC), soil organic matter (SOM), and BC were analyzed. Samples were divided into three classes according to BC content (low, medium, and high BC content) and scanned in the 350–2500 nm range. A support vector machine (SVM) was used to develop prediction models of soil properties. Partial least-square with SVM (PLS-SVM) was used to classify samples for screening purposes. Prediction models of soil properties were at best satisfactory (N_tot_: R^2^ = 0.76, RMSE_CV_ = 0.59 g kg^−1^, RPIQ = 0.65), due to large kurtosis and data skewness. The RMSE_CV_ were large (16.86 g kg^−1^ for SOC), presumably due to the limited number of samples available and the wide data spread. Given our results, the VIS-NIRS method seems efficient for classifying soil samples from an industrialized area according to BC content level (training accuracy of 77% and validation accuracy of 81%).

## 1. Introduction

Black carbon (BC) constitutes a significant part of the soil organic carbon (SOC) stock and plays a crucial role in the global C biogeochemistry. It refers to a wide range of thermally altered carbon-rich (>60%) materials, ranging from char, charcoal, and graphite to highly condensed soot [1]. The type of BC directly depends on the processes of its formation. Soot-BC arises as a result of the re-condensation of volatiles to highly graphitized forms, whereas the solid residues create char-BC [2,3,4]. Soot-BC forms by mutual bonding of small C molecules released by pyrolysis and subsequently recombining by free radical reactions [2] while char-BC forms during the combustion of organic materials with high oxygen access accumulated on solid fuel surfaces.

The formation processes directly affect BC distribution in the environment and thus, soot-BC can be transported over large distances whereas char-BC is mainly deposited at a short distance from burning sites [5,6]. BC’s production dramatically increases yearly due to deforestation, inadequate management of agricultural and forest land, burning of solid fuels, and high levels of low and industrial emissions. Natural fires and technogenic wastes formed during the combustion of wood and fossil fuels are generally considered the main sources of BC [4,7]. The annual volume of BC release into the atmosphere due to fires is estimated at 50 to 270 Tt [8,9].

BC is generally an inert SOC fraction resistant to biological and chemical decomposition and may have a significant residence time, estimated at 2000 [10] or even 5040 years [11,12]. The stability and therefore, the longevity of BC are either due to chemical recalcitrance resulting from its aromatic/clustered structure or to physical protection by minerals or other organic particles (aggregates inclusion) [13,14,15].

Environmental risk related to the presence of BC can be attributed to its occurrence as the main component of suspended dust particles (PM 2.5 and PM 10) which pose a high human health risk [16]. BC shows a high affinity mainly to non-polar substances, particularly planar aromatic compounds such as mono- and polycyclic aromatic hydrocarbons because of its high surface to volume ratio [17]. Aromatic hydrocarbons can be bound to BC by occlusion inside pores and by surface adsorption [18], which causes the fate and bioavailability of these pollutants to be mainly controlled by BC deposited in soil [19,20,21,22]. Additionally, both BC and hydrocarbons are formed in the same high-temperature combustion processes, therefore their presence in industrial areas is strongly correlated. Moreover, BC, due to its high sorption capacity, can also bind trace elements and, as their main carrier, determine their deposition and residence time in the environment [2]. Due to the high ability of BC to bind toxic compounds and the formation of aerosol particles in industrial emission processes capable of crossing cell membranes, the presence of BC in the environment should be subject to constant detection and monitoring.

Generally, the studies on BC sinks and turnover can be associated with diverse environmental aspects ranging from fire histories to global C flux. Different detection and analysis methods are needed to answer questions related to these environmental events and to measure BC amounts and properties from different combustion sources. One of the most popular methods of BC detection in soils is the application of the chemothermal oxidation (CTO-375) technique [13,20]. It involves the thermal oxidation and volatilization of organic carbons at 375 °C, leaving BC residues to be analyzed for radiocarbons [2,12,17,23,24] or to be quantified with carbon elemental analysis [13,17]. Nevertheless, the elementary laboratory analyses for the estimation of BC content are time-consuming, relatively expensive, and cannot describe the spatial and temporal dynamics of BC. Therefore, there is an urgent need for a rapid and accurate approach to screening and measuring BC content over large areas with sufficient detail. The available methods for determining the content of BC in soils and sediments take advantage of the high chemical resistance of BC to degradation. Therefore, the spectroscopic methods could be used as an alternative. It was predicted [2] that the future of BC studies will rely on noninvasive methods of investigation. So far, applications of visible and near-infrared (VIS-NIR) spectroscopy that rely on the light-absorbing properties of soil BC are inexistent. However, mid-infrared (MIR) spectroscopy was once employed successfully [25] to predict BC content in soils collected from several Mollisol areas of the world. The MIR data allowed the authors to classify BC according to its degree of aromaticity and condensation of carbon rings. Diffuse reflectance infrared Fourier transform (DRIFT) technology in the MIR region has a spectral response linearly related to SOC, and this relation was used to estimate charcoal content [26].

Absorption in the VIS-NIR region is dominated by molecules that contain strong bonds between light atoms (e.g., C-H, N-H, C=O, or O-H bonds). The radiation is absorbed in accordance with the concentration of compounds containing these molecules. Therefore, that spectral region is particularly useful for analyzing forms of carbon or nitrogen. VIS-NIR spectroscopy is a still developing technology that allows building databases for the effective identification of soil components through the presence of a characteristic pattern of absorption/emission spectra. Thus, VIS-NIR spectroscopy is known as a non-destructive, rapid, qualitative, and quantitative technique that can provide spectral data both in the laboratory and in the field [27,28]. Moreover, the VIS-NIR method has great potential for simultaneous estimation of a variety of soil properties including, e.g., cation exchange capacity, pH, exchangeable bases, extractable phosphorus, soil texture [29,30], or presence of polycyclic aromatic hydrocarbons [31]. However, most of the studies focused mainly on total SOC concentration [28,32,33,34].

The objective of this study was to investigate the applicability of VIS-NIR spectroscopy to assess the content and properties of BC in soils and the method’s suitability as a fast technique for BC content screening. The research was carried out in an area subjected to strong industrial anthropopression, where a differentiated content of BC from various emission sources was expected (industrial emission and emission as a result of combustion in individual heating furnaces, the so-called low emission). In addition, the area was used for agriculture, therefore the combustion (burning) of plant biomass was also a potential source of BC. Similar research has not been carried out in the country so far, as it is an innovative approach. There is a lack of literature data on this subject. Moreover, the application of the VIS-NIR method to assess the BC content in natural soil samples is also new in soil study, which can facilitate the rapid assessment of risk assessment related to the presence of BC in the soil environment.

## 2. Results and Discussion

### 2.1. Soil physical and Chemical Properties

The presented study was a part of a larger investigation [35,36]. Statistics and correlation matrix of soil properties are provided in Table 1. Detailed statistics for each soil type are provided in Table S1. Soil properties are typical for soil cover from southwest Poland [35,37,38,39] with SOC ranging from 6.97 to 187.16 g kg^−1^. The BC content ranged from 0.23 to 45.29 g kg^−1^. Muck samples containing higher amounts of SOC exhibited higher BC content as compared to sandy soils. Soil BC data are still scarce, especially for Central and Eastern Europe [35]. Therefore, it was impossible to compare our results with other studies from that region. The BC values found here are similar to those obtained from UK urban areas [40] but lower than BC content for urban soils from northeast England [41]. These authors also discovered the increase of BC/SOC ratio with depth suggesting enhanced carbon storage in urban soils. There are several significant correlations (Pearson, α = 0.05) of BC with C_tot_, SOC, SOM, and N_tot_ but no significant correlation between SOC and soil texture was observed. The opposite (significant correlation between SOC and texture) is usually noticed in the literature [28,42]. The soil samples, according to USDA classification, are sands (*n* = 5), sandy loam (*n* = 21), loamy sand (*n* = 14), and muck (*n* = 3) samples. Removing the three muck samples increased the correlation between BC and the other soil properties but also engendered a new significant correlation (α = 0.05) between Ctot, SOC, SOM, and BC/SOC and a higher significant correlation between BC/SOC and clay (Table S2). It was thus decided that these organic samples (mucks) cannot be compared with mineral soils in regard to BC content and thus were removed from the calibration dataset for prediction. The BC/SOC values for the mineral soils are in the range of values found in another study [43], even if the comparison is difficult due to the large discrepancies between analytical methods for BC determination [24].

Few studies have investigated BC content in organic soils. In regard to the BC/SOC ratio, the three muck samples were positioned on the sample distribution margins with the lowest ratios (1.6, 1.9, and 3.5). These mucks were peat deposits that have been drained and cultivated and present BC/SOC ratios similar to those found for several peat sites in China [44]. The variations in BC/SOC ratio were the result of the predominant vegetation according to these authors. BC is a fly-ash compound. Therefore, the BC/SOC values should decrease with increasing distance from the main emitters. This is the case here described in the Materials and Methods section), where high BC/SOC ratios were found near the industrial area and cities. This was also observed for forest soils in Germany [45].

### 2.2. Spectra Description

**Figure 1:**
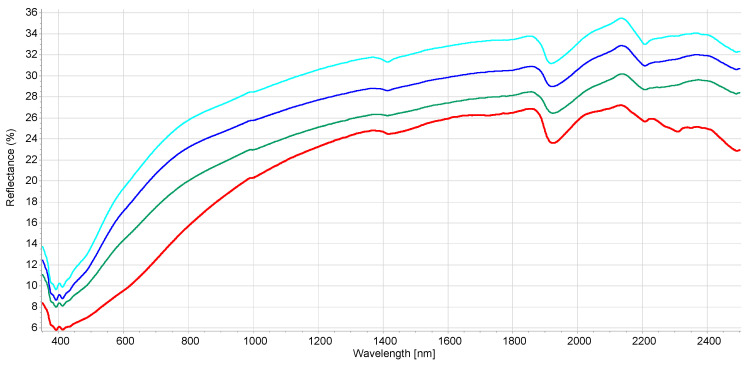
Mean reflectance spectra for the soil class texture. Red—muck samples, green—sand, light blue—sandy loam, dark blue—loamy sand (according to USDA classification).

**Figure 2:**
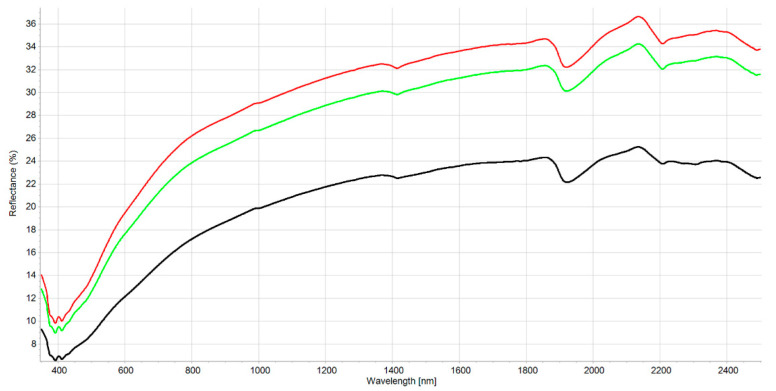
Mean reflectance spectra for the three classes of the PLS-SVM classifier. The three classes of BC content are represented low (0–0.7 g kg^−1^) as green line: medium (0.71–2.0 g kg^−1^) red line and: high (>2.01 g kg^−1^) black line.

The interpretation of the VIS-NIR spectrum is extremely complex because it is the result of overtones and combinations from primary absorption in the mid-infrared region with many overlapping peaks and valleys. Consequently, no distinct or well-defined peaks are noticeable and, e.g., it is impossible to point out in the spectrum where BC is. Many factors affect soil spectroscopic properties [46] (soil chemistry, physics, or biology). It was demonstrated that other external factors (e.g., land management [29]) but also amendment with biochar [22] can modify the soil spectrum. Figure S1 shows the spectral variability of the dataset after the moving average (MA) transformation. Figure 1 and Figure 2 represent the mean spectra for the four soil groups (sand, loamy sand, sandy loam, and muck) and the three classes of BC content, respectively. It was observed (1) that a higher sand content was usually associated with a higher reflectance due to light scattering decreasing with larger particle size, and (2) a higher BC content was associated with a lower reflectance due to the absorption of light by the -C bonds The moving average reduced random noise. All spectra are similar in appearance except for muck samples. In the visible region (350–700 nm), most of the differences are the result of sample color caused by organic matter and iron oxide presence. The peaks at 390–410 nm and the change of curvature at around 450 nm are probably due to Fe oxides [46] and humic acids [47]. The main differences between samples are the baselines and the reflectance intensity at 1410, 1920, and 2200 nm absorption peaks related to O-H bonds of bound or hygroscopic water [48]. The reflectance of the 2200 nm region is also related to clay minerals (Al-OH bend plus O-H stretch) of kaolinite and gibbsite [49]. The highest reflectance was observed at 2135 nm from the N-H and C=O combination region [50]. This is in accordance with the finding that the maximum correlation between raw reflectance spectra and soil organic matter is found at 2137 nm [51]. The flattest spectra with the lowest reflectance were samples (mineral soils) with high C and N content since organic matter tends to decrease the overall reflectance [52]. The mucks (in red) presented differences with mineral soil spectra, especially in the 1900–2500 nm range (combination band region). This is due to light absorption by organic functional groups (decayed plant material in mucks) and also to sample surface roughness [53]. According to some researchers [51], the first derivative is the most suitable pre-processing to study soil organic matter. In our case, prediction models were not improved using the first derivative. The models were less robust. Therefore, the presented results are for raw spectra with moving average only. Nevertheless, the first derivative is suitable to remove the baselines and the spectral features (peaks) are more visible. Figures S2 and S3 present the first derivatives of polynomial order 2 with 11 smoothing points for the mean spectra of the four soil groups and the three levels of BC content, respectively. The spectrum of soils with high BC content (Figure S3) is most similar in shape to the spectra of low and medium BC content but with lower intensities in the visible region (350–780 nm) due to higher light absorption probably caused by higher BC content. The difference in absorption is lowering until 780 nm and the beginning of the NIR region. The large peaks in the 350–450 nm range are the result of noise enhancement caused by the differentiation of the derivative [54]. In the 780–1300 nm, the first derivative spectra are very similar for all BC content. After that, several regions and peaks for the high BC content differ from low and medium BC content (e.g., lower reflectance for high BC at 1420, 1725, and 2188 nm which is probably due to absorption by aromatic hydrocarbons and C-H Aryl [50]). This could indicate PAH absorption by the soil BC. This is similar to results from another study showing absorption of PAHs in soils with higher BC content [35]. The derivatives from muck samples are also very different from mineral soils in that spectral region (combination band region). Some enlargements of the first derivative of these different regions are provided in Figures S4–S6.

### 2.3. PCA Analyses

**Figure 3:**
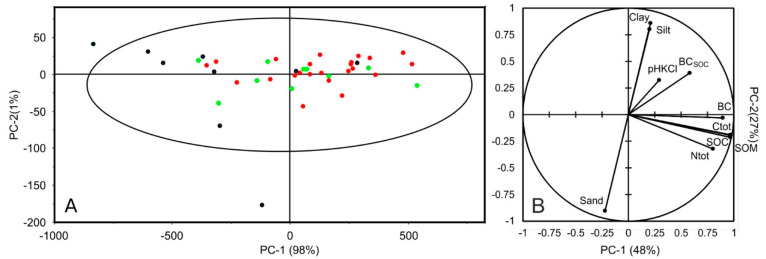
PCA scores with Hotelling T2 ellipse (PC-1 and PC-2) from the PCA application to soil spectra (**A**) and principal plane (PC-1 and PC-2) of the investigated soil properties (**B**). The three classes of BC content are represented as green dots—low (0–0.7 g kg^−1^); red dots—medium (0.71–2.0 g kg^−1^) and black dots high (>2.01 g kg^−1^).

Figure 3A shows the Hotelling ellipse (95% confidence interval) resulting from the application of a PCA to the raw soil spectra (350–2500 nm). The first two components account for 99% of the observed variance. The samples are distributed along the PC-1 axis (98% of explained variance) with two samples (mucks) outside the ellipse. Computing the PCA without the muck samples did not change sample distribution on the plane. No sample grouping was discernable, and no outliers were detected except for the muck sample down the PC-2 axis. Figure 3B presents the application of PCA to soil properties. The first component (PC-1) is associated with properties related to organic functional groups (C and N) and the second component with soil texture. This presents similarities with soil samples from Brittany [55]. BC is not correlated to soil texture with regards to Figure 3B but seems correlated with C_tot_, SOC, and SOM.

### 2.4. Prediction of Soil Properties

Prediction results from the SVM regression are presented in Table 2. After a preliminary examination of the results, raw data with MA was used for building models since there was strong overfitting of the SVM regressions (R^2^ > 0.90) in the calibration step with the pre-processed spectra with rather poor R^2^ for the validation step. Pre-processing the spectra does always improve the modeling. Raw spectra were also used with success [56] to predict SOM from reclaimed soils after coal mines with PLS-SVM regression. Here, the best predictions were obtained for N_tot_, C_tot_, and SOM but the parameters of the prediction (0.61 < R^2^ < 0.76 with RPIQ < 1) fall short with regard to what can be found in the literature [30,34]. The SVM regression for SOC shows a very high RMSECV and a low RPIQ. SOM uses to be an estimate of SOC but the results of the SVM regression are not similar despite a very high correlation (0.983, α = 0.05). SOM needed 25 support vectors while SOC needed 12 support vectors to complete the regression. With SVM, the prediction relies only on support vectors which are a subset of the original samples that are outside the boundary given by the model. The 13 supplementary support vectors are mostly from the samples with the highest clay content (<0.002 mm). It is possible that SOM prediction was also based on clay content since clay and organic matter are complexed as complexed organic carbon (COC) [57]. Moreover, with chemical oxidation methods, some hydrophobic compounds (organic matter) are not attacked or oxidized. This could explain the differences in prediction results for SOM and SOC. Even if sometimes pH can be predicted by the method thanks to indirect spectral responses, the poorest prediction was obtained for pH_KCl_ (R^2^ = 0.22). Similar results for pH prediction on oven-dry or field-moist soils in Missouri (USA) [58] were obtained (R^2^ = 0.02 and R^2^ = 0.03) despite good or satisfactory models for many other soil properties. Sand and silt models presented acceptable RPIQ (2.08 and 1.87 respectively) but with R^2^ < 0.5. Despite these poor models, the RPIQ are quite reasonable. From these results, some remarks can be made. Only sand and silt presented a normal distribution, but the non-normal distribution of the other properties did not affect the SVM regression since the method does not require a normality assumption. Nevertheless, we tried to transform the data to obtain normality, but it did not improve the prediction results (not presented here). The RPD clearly depends on the sample distribution and could mislead the interpretation of a model if taken alone. RPD responds very much to the standard deviation (SD). If the SD is large, the RPD will increase despite the weak prediction model. Of course, large datasets are less subjected to that rule. This was already pointed out [27] with the suggestion to use to use RPIQ instead since it is not subjected to the skewness of the distribution. Higher RPIQ indicates better robustness of a model. For BC/SOC, the RPIQ is 18.68. Given our results, RPIQ is not suitable with small datasets that are skewed and present large standard deviations. It is noticed that models tended to give an underestimate of SOC and SOM values in the highest range of these properties content. This phenomenon was also observed for the prediction of C_tot_ and N_tot_ content [59]. It is clear that in the present case, it is due to the poor representation of samples with high SOC and SOM values in the dataset. During the cross-validation, the samples in the validation dataset can have values that are not represented in the calibration dataset, and thus the SVM regression is forced to predict samples outside the range of the model.

The present dataset seems not to be very numerous for building a robust VIS-NIR model, but this work is of preliminary nature. Nevertheless, 40 samples were used in several other studies [29,60,61] with success for SOC in particular and other soil properties prediction. In the case of on-the-go mapping with a mobile VIS-NIR sensor, even 15 samples are enough for calibration [62] but at the field scale and hence with less soil variability.

### 2.5. Prediction of BC Content and Attempt to Use the VIS-NIR Spectra for Screening Purposes

Since biochar can significantly modify soil spectra [22], it was hypothesized that BC is a suitable material for VIS-NIR prediction, especially since we found a good correlation between BC and C_tot_, SOC, and SOM (Table 1, Figure 3B) and the fact that these properties are usually well predicted by the method. VIS-NIR calibrations are only valid if the reference data are correct and accurate. In the present work, the CTO 375 method was used to determine BC content. The method CTO 375 is considered robust for BC quantification in soils even in presence of high non-pyrogenic SOC and carbonate contents [23]. These authors also demonstrated the variations of BC content along soil profiles. This leads us to believe that the VIS-NIR, a fast, cheap, and easy-to-implement method could be suitable for predicting BC content or at least classifying samples with low, medium, or high BC content and probably in the future to describe SOC and BC variations and other soil properties along profiles [26,63,64].

The results obtained for BC content (R^2^ = 0.26, RMSECV = 7.18) and BC/SOC (R^2^ = 0.27, RMSECV = 0.26) predictions were poor. The reason why, despite the significant correlation between C_tot_, SOC, SOM, and BC, the prediction models for BC content being not robust enough, is that the light in the NIR region is absorbed by chemical bonds (e.g., C-H, C=O, O-H) of the organic functional groups that are present in any type of BC and that traditional methods of BC determination are measuring only one type of BC. The NIR spectra are on the other hand the result of light interaction with the sum of BC in the soil and with the other forms of C (C_tot_ and SOC). Thus, the model predicts the total amount of BC and not only one type of BC as the reference data. Nevertheless, the unsuccessful BC prediction could be due to the small number of samples in the dataset and the skewed data distribution. Therefore, the cross-validation was not able to correctly predict samples on the margin of the distribution. This was observed elsewhere [31] for the prediction of polycyclic aromatic hydrocarbons. In the future, and for improving prediction results, building a larger spectral library covering a wider range of BC values with ideally a uniform distribution will be needed to check the screening method on other soil groups. After using the adequate correction method to correct for soil moisture, one could measure the level of BC content in the field, the PLS-SVM algorithm being efficient in these conditions [65].

**Figure 4:**
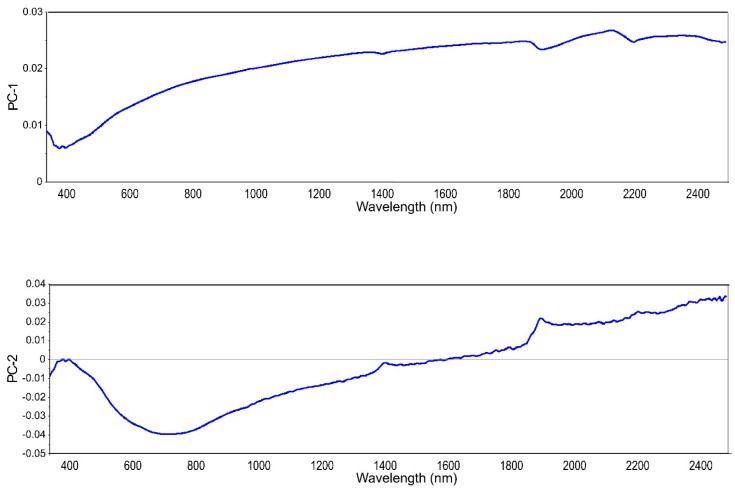
Loading weights for the first two principal components PC-1 and PC-2.

Despite the lack of a robust model for BC content prediction, we decided to use those samples in an attempt to determine the level of soil BC. Being able to quickly determine the level of soil BC could be a first step to deciding whether or not further investigations are needed. Therefore, a PLS-SVM classifier was applied to group samples according to their BC content. The three class were: low—0–0.7 g kg^−1^, medium—0.71–2.0 g kg^−1^, and high—>2.01 g kg^−1^. There were 10 samples of the low class, 20 samples of the medium class, and 10 samples of high class. Figure 4 shows loading weights for the first two principal components from the PLS regression on raw spectra with MA for the three BC classes. These two components accounted for 98% of the variation. The shape of PC-1 loadings reminds of a mineral soil reflectance spectrum without marked features. PC-2 was the suggested PC by the software (based on the residual variance curve) for use in the PLS modeling. However, here we only needed the values of the principal components for further investigation with SVM. The SVM algorithm was used on the first ten components with the three classes as a category to obtain the PLS-SVM classification. The training accuracy was 77% and the validation accuracy was 81%. The classifier had more difficulties correctly classifying samples with a low BC content. This is due to the lack of spectral features for those samples.

There are still large discrepancies between methods as Schmidt et al., [66] demonstrated by investigating eight soil samples for BC content by six techniques. These discrepancies were often due to the absence of a clear definition of what is BC and the lack of certified BC material.

As stated elsewhere [43], there is an urgent need to improve BC methods of analysis as BC contents in soils are hardly comparable between studies. Moreover, the detection of BC is of importance since it was demonstrated that it has a higher sorption capacity than natural organic matter [67]. Furthermore, processes of latter potentially harmful contaminants released from BC are still not well understood [68]. From our study, it is clear that at the moment, reflectance spectroscopy is not a suitable method for the precise measurement of BC in the soil. However, the method appears very promising for the screening approach. The method is fast, not expensive, and does not require the use of chemicals or very high temperatures. In regard to our results, the method could be used as a tool for rapid screening and mapping. The main appeal of the method is that it can be employed in the field. In the future, a soil spectral database with information on BC content could probably help to improve the prediction ability of the method, since information on BC seems included in the VIS-NIR spectra. Quantitative modeling of BC content with reflectance spectroscopy failed, probably due to insufficient representation of samples in the margins of the distribution since properties usually well predicted were unsuccessfully predicted. The division into three classes, though arbitrary, due to a lack of international guidelines allowed the classification of soil according to its BC content low—medium—high. This is useful in practice since most contaminants are readily adsorbed on BC molecules. The paper is a starting point for further improvement of the technique.

## 3. Materials and Methods

### 3.1. Site Description and Soil Samples Collection

Forty-three samples (about 1 kg each) covering a wide range of physicochemical properties were taken from the surface layer (0–20 cm) of agricultural soils. Soil materials were transported to the laboratory, air-dried for 48 h at a temperature of about 20 °C, well-mixed, sieved through a 2 mm mesh sieve, and stored in the dark in a glass jar.

**Figure 5:**
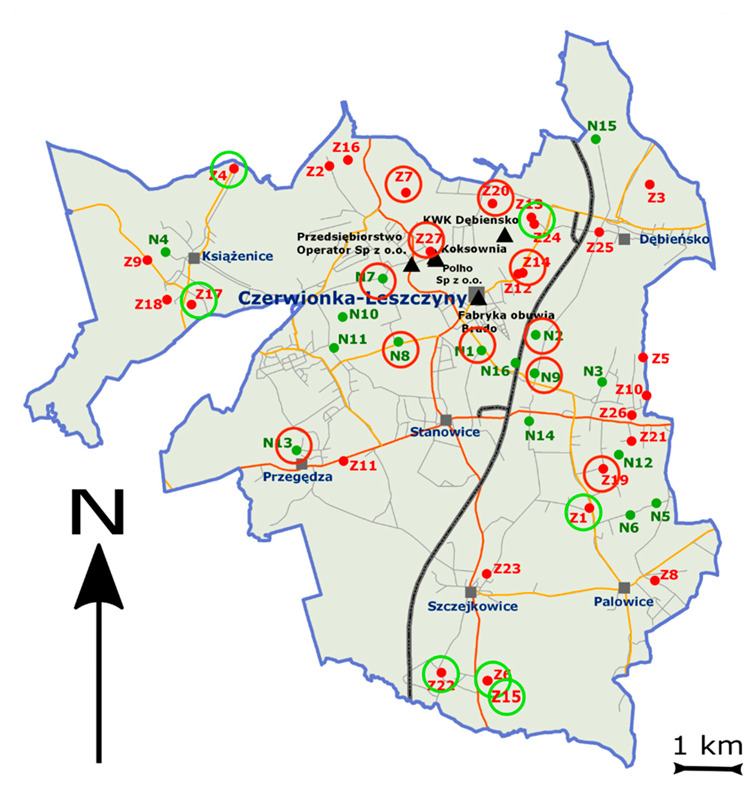
Sample localization (N and Z)—red circles at locations with high BC/SOC (>10) and green circles at a location with low BC/SOC (<5). The red color (Z points) for areas with PAH contamination and the green points (N) for the areas with no PAH contamination. The black triangles are the main sources of BC emission.

The environmental studies included soils collected from areas subject to long-term and strong industrial anthropopressure (Figure 5) exposed to the accumulation of BC. Based on the IUNG-PIB databases, the research sites were located in the Czerwionka-Leszczyny commune, located in the Rybnik poviat, Śląskie voivodship. The soils from this area were the subject of previous studies carried out by IUNG-PIB [22,35,36].

The Czerwionka-Leszczyny commune is part of the western subregion, which is geographically located between the upper Silesian industrial district and the Karviná-Ostrava industrial district. The main causes of soil pollution in this area include the emissions from point energy sources related to the combustion of solid fuels, traffic pollution, and the impact of other industrial plants. In addition, over many years, the mining industry located in the studied area and the neighboring communes, i.e., the Knurów-Szczygłowice, Dębieńsko, Budryk, and Łaziska coal mines, was the most burdensome for the environment. The result of many years of operation of mines and the influence of the Rybnik industrial district is a strong transformation of the area resulting from intense mining activity, related to the presence of numerous heaps and post-mining waste dumps.

The soils in the commune covered by the research are of poor quality. Sandy soils, formed of slightly loamy sands, dominate. Brown (acidic and leached) and podzolic soils have the largest share of the overall structure of the land.

### 3.2. Determination of Soil Properties

The samples were analyzed for texture, pH_KCl_, total nitrogen (N_tot_), and total carbon (C_tot_) content as well as soil organic carbon (SOC) and soil organic matter (SOM). Soil texture was determined by the aerometric method (PN-R-04032, 1998). The pH was measured potentiometrically in a 1:2.5 (m·V^−1^) suspension of soil in 1 (mol∙L^−1^ KCl solution) (PN-ISO 10390, 1997). A Vario Macro Cube CN Elementar Analyzer (Elementar Analysensysteme GMBh) was used to determine C_tot_ and N_tot_ by thermal conductivity detection after oxidation of soil C to gaseous reaction products. SOC content was determined by sulfochromic oxidation of organic carbon (PN-ISO 14235, 2003) followed by titration of the excess K_2_Cr_2_O_7_ with FeSO_4_(NH_4_)_2_SO_4_·6H_2_O (Tiurin method). The samples for SOM analysis were incinerated in a furnace at 550 °C (loss-on-ignition (LOI) method). All methodologies are described in detail elsewhere [35].

### 3.3. Determination of Black Carbon

Thermal analysis of BC (CTO-375 method) was used to determine BC in soils [20]. The method essentially consists of three distinct steps. Step one: the determination of carbonates by chemical treatment after their removal from soil samples. Samples were acidified with 10% HCl and the content of CaCO_3_ was calculated from the volume of carbon dioxide emitted, using the Scheibler apparatus [69]. Step two: thermal treatment to remove non-BC organic carbon from soil samples. Two gram portions of soils were combusted at 375 °C for 16 h in a muffle furnace with oxygen supply. Step three: determination of BC residue with C-N analyzer (Vario Macro Cube CN Elementar Analyzer, GMBH, Langenselbold, Germany). To compare the obtained results with other studies, the percentage of BC in relation to SOC (BC/SOC) content was calculated.

### 3.4. Spectroscopic Measurements

Before spectral analysis, soil samples were oven-dried at 45 °C for 12 h. The samples were allowed to cool down in the oven and then scanned in the 350–2500 nm spectral range using a portable VIS-NIR spectroradiometer PSR-3500^®^ (Spectral Evolution Inc., Lawrence, MA, USA). The instrument has spectral resolutions of 3.5 nm at 700 nm, 10 nm at 1500 nm, and 7 nm at 2100 nm, respectively, and sampling intervals of 1.5 nm at 700 nm, 3.8 nm at 1500 nm, and 2.5 nm at 2100 nm. Soil reflectance was interpolated to 1 nm intervals. Samples, placed in a Petri dish, were scanned using a contact reflectance probe featuring a 5W built-in light source. The contact probe allowed full contact with the sample, thus avoiding outside interference. The spectroradiometer was calibrated using a 99% white NIST reference panel (5 × 5 cm). Four replicate scans were taken for each soil sample, one scan for each of the four quadrants of the Petri dish. Each scan was an average of 30 scans, where the averaging was performed by the instrument. The spectrometer was recalibrated after every five soil samples using a white reference.

### 3.5. Spectra Pretreatments and Model Construction

The whole spectra were used for modeling since no significant noise was observed along the 350–2500 nm range. The following mathematical and statistical treatments were applied to the spectra using Spectragryph software: moving average (MA) for smoothing, and then first and second Savitzky–Golay derivatives (SG-D), standard normal variate (SNV), multiplicative scatter correction (MSC), and detrending. Information about pre-processing techniques and aims can be found elsewhere [70]. Principal component analysis (PCA) was applied on raw spectra with MA to explore spectral variability and also on the investigated soil properties to identify relations between those properties. PCA and correlation matrices were computed with XLSTAT 19.3 (Addinsoft, Paris, France). Support vector machine (SVM) regression with leave-one-out cross-validation was used to correlate spectral data with reference data (soil properties) to obtain prediction models for all investigated properties. SVM is a regression method based on statistical learning with no assumption for normality and is known to work better than other methods on unbalanced data [71]. Model quality was evaluated by using the R^2^ value (references vs. predicted values), root mean square error of cross-validation (RMSECV), the ratio of standard deviation (RPD), and the ratio of performance to interquartile range (RPIQ).

### 3.6. Classification of BC Content

To use the VIS-NIR method as a fast screening device, the BC content from laboratory data was divided into three classes (low: 0–0.7 g kg^−1^; medium: 0.71–2.0 g kg^−1^; high: >2.01 g kg^−1^) defined in regards to our BC dataset and which are in accordance with the literature [1,40]. A combination of partial least-square (PLS) and SVM classification was applied to the spectra to classify soil samples according to the BC classes. The SVM classifier was run on the Y-scores of PLS regression for dimension reduction purposes. Y-scores of the 10 first components were obtained by applying the PLS1 algorithm on two matrices (soil spectra and classes of BC content). All pretreatments and calibrations were performed with Unscrambler X^®^ 10.3 (Camo AS, Oslo, Norway) on mean-centered data.

## 4. Conclusions

This paper reports the first attempt at using visible and near-infrared spectroscopy to investigate BC content in agricultural soils from an industrialized region. Robust models for the prediction of BC content were not possible to obtain with the present dataset; however, it was demonstrated that the method shows promise as a tool for rapid soil screening. It was possible to classify soil samples according to BC content levels (low, medium, high) with a validation accuracy of 81%. In the future, a larger soil spectral database with more soil types should be used to develop more robust models of BC content prediction to improve accuracy. That could allow the use of the method instead of the more expansive and time-consuming regular methods of BC determination. This should also further improve classification accuracy.

## Figures and Tables

**Table 1:** Summary statistics of soils samples and correlation matrix of soil properties (*n* = 43).

	Sand	Silt	Clay	Ctot	Ntot	SOC	SOM	BC	BC/SOC	pH_KCl_
**Min.**	49.00	9.00	0.00	8.48	0.76	6.97	19.89	0.23	1.59	3.80
**Max.**	90.00	45.00	6.00	202.68	11.67	187.16	412.07	45.29	38.51	7.80
**Mean**	71.61	26.68	1.71	30.80	1.84	26.98	63.57	2.48	9.31	5.15
**SD**	9.41	8.48	1.72	44.19	2.00	39.08	80.08	6.80	7.22	0.93
**Sand**	**1**									
**Silt**	**−0.987**	**1**								
**Clay**	**−0.606**	**0.470**	**1**							
**Ctot**	−0.031	−0.016	0.248	**1**						
**Ntot**	−0.007	0.012	−0.018	**0.841**	**1**					
**SOC**	−0.016	−0.038	0.272	**0.987**	**0.836**	**1**				
**SOM**	−0.058	0.016	0.233	**0.982**	**0.913**	**0.979**	**1**			
**BC**	−0.078	0.043	0.212	**0.768**	**0.494**	**0.671**	**0.673**	**1**		
**BC/SOC**	−0.268	0.227	**0.348**	0.287	0.060	0.200	0.232	**0.627**	**1**	
**pH_KCl_**	−0.208	0.231	0.000	0.013	−0.039	−0.045	−0.006	0.254	**0.543**	**1**

Sand, silt, clay (%, USDA classification), C_tot_ total carbon content (g kg^−1^), N_tot_ total nitrogen (g kg^−1^), SOC soil organic carbon (g kg^−1^), SOM soil organic matter (g kg^−1^), BC black carbon (g kg^−1^), BC/SOC ratio, SD standard deviation. Values in bold are different from 0 with a significance level α = 0.05.

**Table 2:** Results of the cross-validated prediction for the investigated soil properties using SVM regression.

Soil Properties	R^2^	RMSECV	RPD	RPIQ
**Sand**	0.49	6.73	1.41	2.08
**Silt**	0.41	6.56	1.30	1.87
**Clay**	0.55	1.18	1.35	0.85
**C_tot_**	0.61	25.07	1.15	0.24
**N_tot_**	0.76	0.59	1.65	0.65
**SOC**	0.52	16.86	0.99	0.25
**SOM**	0.63	19.50	1.28	0.37
**BC**	0.26	7.18	0.98	0.12
**BC/SOC**	0.27	0.26	27.74	18.68
**pH_KCl_**	0.22	0.82	1.11	1.22

Sand, silt, clay (%, USDA classification), C_tot_ total carbon content (g kg^−1^), N_tot_ total nitrogen (g kg^−1^), SOC soil organic carbon (g kg^−1^), SOM soil organic matter (g kg^−1^), BC black carbon (g kg^−1^), BC/SOC ratio.

## Data Availability

The raw data presented in this study are available on request from the corresponding author. The data are not publicly available due to intellectual property.

## Supplementary Materials

The following supporting information can be downloaded at: https://www.mdpi.com/article/10.3390/molecules27217334/s1, Figure S1. Reflectance spectra of the 43 soil samples. Red—muck samples, green—sand, light blue—sandy loam, dark blue—loamy sand (according to USDA classification); Figure S2. First derivative of mean spectra for the soil class texture. Red—muck samples, green—sand, light blue—sandy loam, dark blue—loamy sand (according to USDA classification); Figure S3. First derivative of mean spectra for the three classes of the PLS-SVM classifier. The three classes of BC content are represented by low (0–0.7 g kg^−1^) in the orange line: medium (0.71–2.0 g kg^−1^) in the brown line and: high (>2.01 g kg^−1^) black line; Figure S4. Enlargement in the visible range of the first derivatives for BC content levels spectra (from Figure S3). The three classes of BC content are represented low (0–0.7 g kg^−1^) in the orange line: medium (0.71–2.0 g kg^−1^) brown line and: high (>2.01 g kg^−1^) black line; Figure S5. Enlargement (from Figure S3) of a NIR region of the first derivatives for BC content levels spectra; Figure S6. Enlargement (from Figure S3) of a NIR region of the first derivatives for BC content levels spectra; Table S1: Summary statistics for the different soil types; Table S2: Summary statistics of soils samples and correlation matrix of soil properties (*n* = 40, without 3 muck samples).

## Sample Availability

All samples as well as raw data are available from the authors of the publication.
