# Supplementary material for: Diffuse Reflectance Spectroscopy for Black Carbon Screening of Agricultural Soils under Industrial Anthropopressure
Source: Molecules. 2022 Oct 28;27(21):7334. doi: 10.3390/molecules27217334 (PMC9658794; doi:10.3390/molecules27217334)
Supplement: Supplementary file 1 [file molecules-27-07334-s001.zip › molecules-1923434-supplementary.pdf]

SUPPORTING INFORMATION

Diffuse Reflectance Spectroscopy for Black Carbon Screening of Agricultural Soils under Industrial Anthropopressure

Guillaume Debaene [1]*, Aleksandra Ukalska-Jaruga [1], Bożena Smreczak [1], and Ewa Papierowska [2]

1. Institute of Soil science and Plant Cultivation – State Research Institute, Department of Soil Science Erosion and Land Protection, Czartoryskich 8, 24-100 Puławy, Poland
2. Warsaw University of Life Sciences, Institute of Environmental Engineering, Water Centre, Nowoursynowska 159, 02-776 Warsaw, Poland

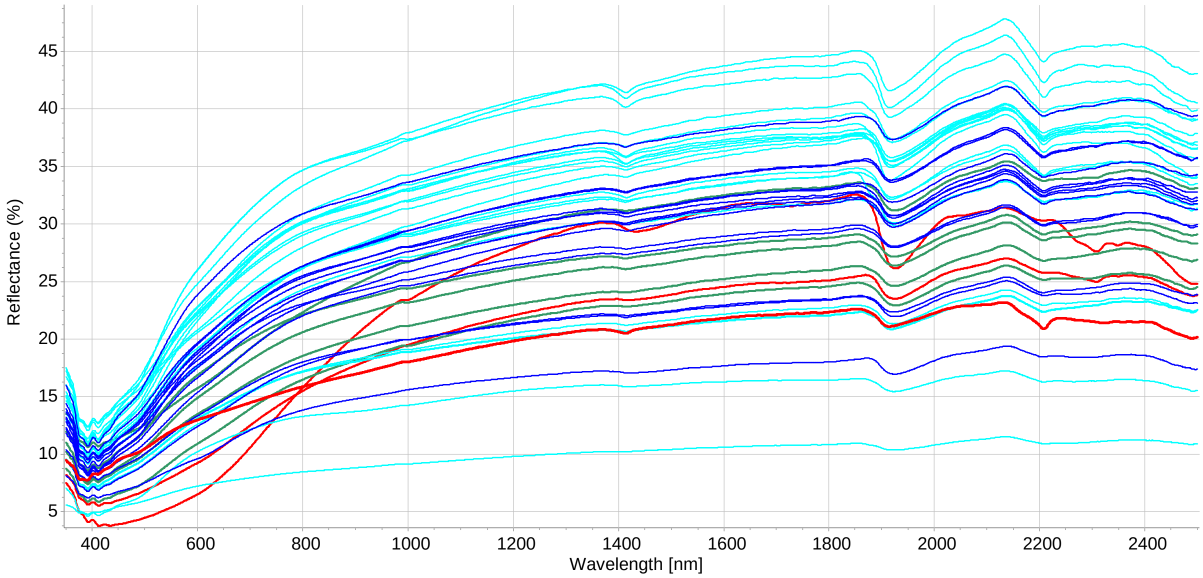

Figure S1. Reflectance spectra of the 43 soil samples. Red – muck samples, green – sand, light blue – sandy loam, dark blue – loamy sand (according to USDA classification).

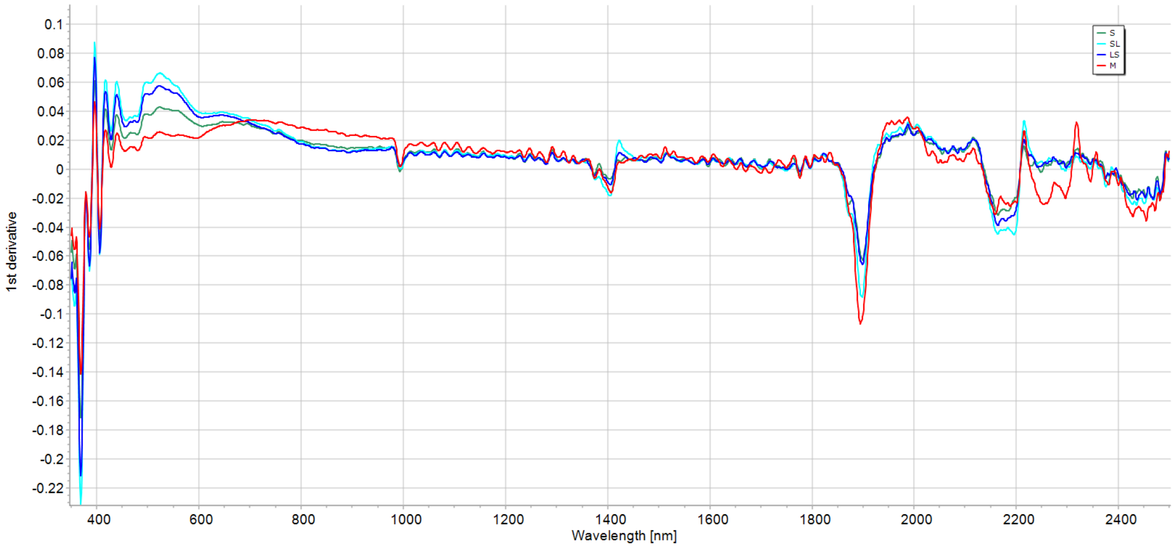

Figure S2. First derivative of mean spectra for the soil class texture. Red – muck samples, green – sand, light blue – sandy loam, dark blue – loamy sand (according to USDA classification).

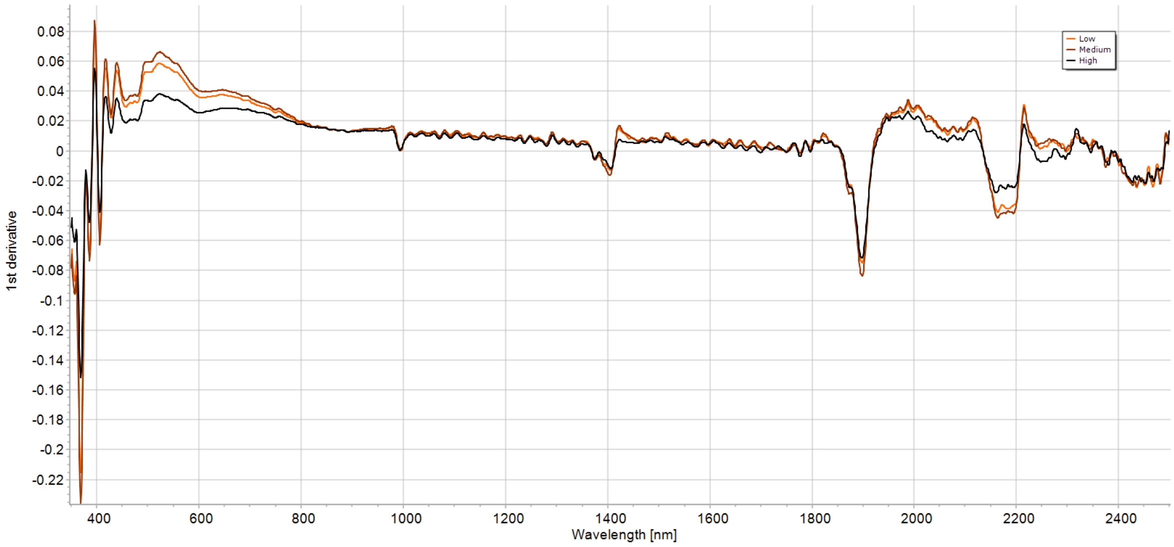

Figure S3. First derivative of mean spectra for the three classes of the PLS-SVM classifier. The three classes of BC content are represented low (0-0.7 g kg^{-1}) as orange line: medium (0.71-2.0 g kg^{-1}) brown line and: high (> 2.01 g kg^{-1}) black line.

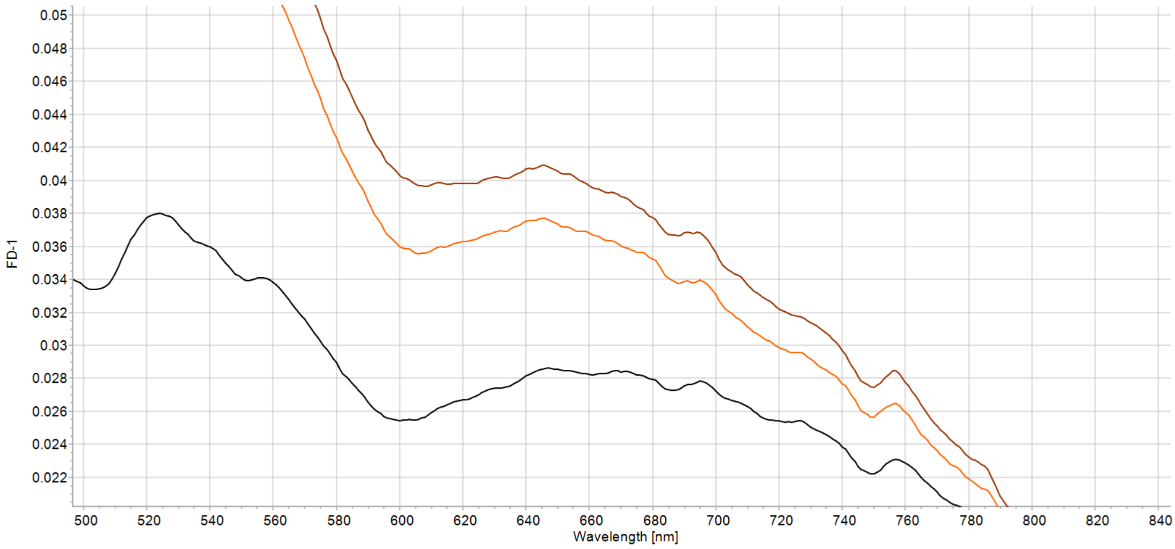

Figure S4. Enlargement in the visible range of the first derivatives for BC content levels spectra (from Figure S3). The three classes of BC content are represented low (0-0.7 g kg^{-1}) as orange line: medium (0.71-2.0 g kg^{-1}) brown line and: high (> 2.01 g kg^{-1}) black line.

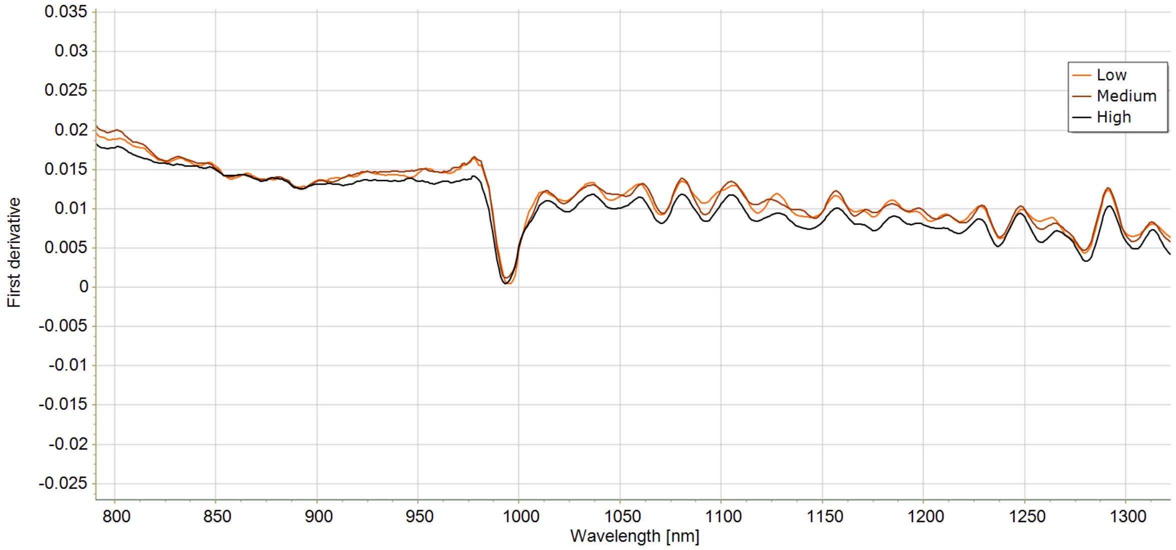

Figure S5. Enlargement (from Figure S3) of a NIR region of the first derivatives for BC content levels spectra.

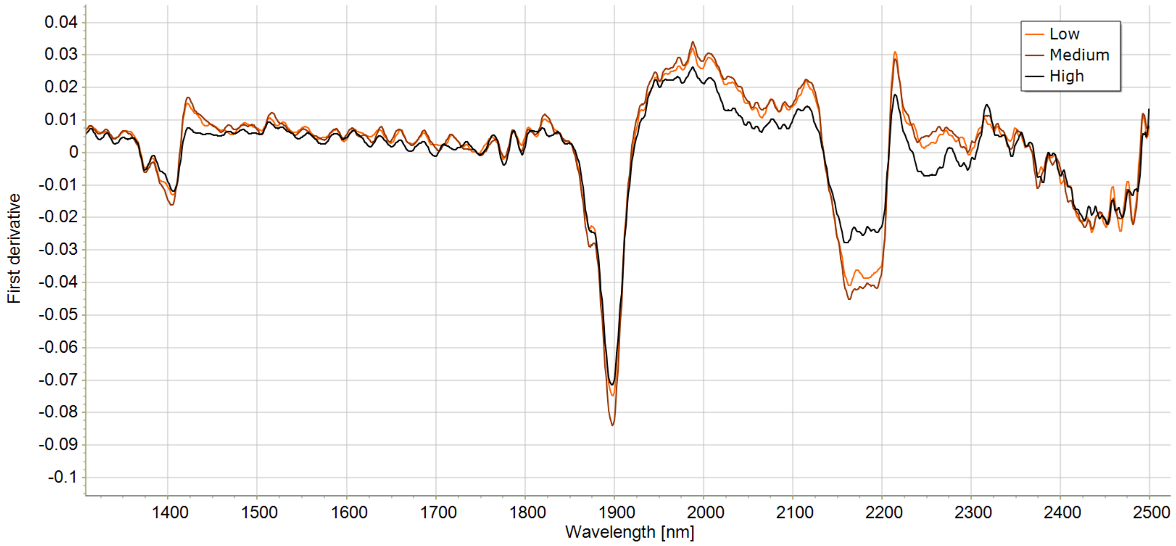

Figure S6. Enlargement (from Figure S3) of a NIR region of the first derivatives for BC content levels spectra.

Table S1. Summary statistics for the different soil types.

	Sand	Silt	Clay	C_{tot}	N_{tot}	SOC	SOM	BC	BC/SOC	pH_{KCl}
					**Mucks**					
Min.	-	-	-	117.90	3.57	106.73	220.25	2.06	1.59	3.80
Max.	-	-	-	202.68	11.67	187.16	412.07	3.70	3.46	4.30
Mean	-	-	-	149.59	7.35	141.30	290.51	3.08	2.30	4.07
SD	-	-	-	26.71	6.81	23.89	61.03	0.51	0.59	0.15
					**Sand**					
Min.	80	9	0	9.99	0.79	8.87	23.74	0.23	2.02	4.00
Max.	90.00	20.00	1.00	22.68	1.68	20.86	46.76	1.54	8.77	6.10
Mean	87	12.50	0.50	15.68	1.22	13.89	33.40	0.77	5.64	4.90
SD	1.51	1.59	0.22	1.99	0.14	1.82	3.86	0.17	1.05	0.32
					**Sandy loam**					
Min.	49.00	26.00	1.00	9.06	0.82	7.96	26.94	0.50	5.07	4.00
Max.	73.00	45.00	6.00	167.25	4.78	124.82	240.65	45.29	38.51	7.30
Mean	63.81	33.25	2.94	27.96	1.45	22.56	56.63	4.37*	12.95	5.42
SD	1.48	1.25	0.42	10.88	0.29	8.17	16.17	2.76	2.49	0.26

				**Loamy sand**						
Min.	62.00	16.00	0	8.48	0.76	6.97	19.89	0.64	4.85	3.80
Max.	83.00	38.00	2.00	56.18	4.64	48.12	139.19	3.67	19.21	7.80
Mean	73.28	25.94	0.78	18.56	1.46	16.21	41.96	1.27	8.47	5.18
SD	1.27	1.24	0.15	2.96	0.23	2.54	6.91	0.19	0.85	0.20

*The high mean content for BC in sandy loam was caused by one sample with very high BC content (45.29 g kg-1). The median 0.99 is similar to the media (1.01) for loamy sand. SD standard deviation.

Table S2. Summary statistics of soils samples and correlation matrix of soil properties (n = 40, without 3 muck samples).

	**Sand**	**Silt**	**Clay**	**C_{tot}**	**N_{tot}**	**SOC**	**SOM**	**BC**	**BC/SOC**	**pH_{KCl}**
**Min.**	49.00	9.00	0.00	8.48	0.76	6.97	19.89	0.23	2.02	3.80
**Max.**	90.00	45.00	6.00	167.25	4.784	124.82	240.65	45.29	38.51	7.80
**Mean**	71.55	26.85	1.60	21.89	1.421	18.40	46.55	2.44	9.84	5.23
**SD**	9.52	8.51	1.60	28.74	0.974	21.82	45.55	7.06	7.21	0.91
**Sand**	**1**									
**Silt**	**-0.990**	**1**								
**Clay**	**-0.680**	**0.572**	**1**							
**C_{tot}**	-0.060	0.056	0.059	**1**						
**N_{tot}**	-0.022	0.057	-0.173	**0.828**	**1**					
**SOC**	-0.053	0.054	0.029	**0.998**	**0.857**	**1**				
**SOM**	-0.092	0.096	0.035	**0.975**	**0.918**	**0.983**	**1**			
**BC**	-0.078	0.043	0.235	**0.895**	**0.526**	**0.870**	**0.791**	**1**		
**BC/SOC**	-0.265	0.210	**0.464**	**0.444**	0.128	**0.408**	**0.385**	**0.636**	**1**	
**pH_{KCl}**	-0.204	0.208	0.108	0.161	0.044	0.142	0.143	0.260	**0.524**	**1**

Sand, silt, clay (%, USDA classification), C_{tot} total carbon content (g kg^{-1}), N_{tot} total nitrogen (g kg^{-1}), SOC soil organic carbon (g kg^{-1}), SOM soil organic matter (g kg^{-1}), BC black carbon (g kg^{-1}), BC/SOC ratio, SD standard deviation. Values in bold are different from 0 with a significance level alpha = 0.05.
